# Enhancement of growth performance in pre-weaning suckling Boer kids supplemented with creep feed containing alfalfa

**DOI:** 10.14202/vetworld.2015.718-722

**Published:** 2015-06-15

**Authors:** Nay Nang Htoo, Aung Tun Khaing, Yusuf Abba, Nwe Nwe Htin, Jesse Faez Firdaus Abdullah, Than Kyaw, Mohd Azam Khan Goriman Khan, Mohd Azmi Mohd Lila

**Affiliations:** 1 University Malaysia Kelantan, 16100 Kota Bharu, Kelantan, Malaysia; 2 Faculty of Veterinary Medicine, Universiti Putra Malaysia, 43400 UPM Serdang, Selangor, Malaysia

**Keywords:** boer, creep feed, growth performance, pre-weaning, alfalfa

## Abstract

**Aim:**

This study examined the effects of creep feed (CF) supplementation (with or without Alfalfa) on the pre-weaning growth performance of nursing goat kids.

**Materials and Methods:**

A total of forty eight (48), 7 days old, single born kids (live weight 4.4±0.09 kg) were divided into three treatment groups, each containing eight males and eight females. All three groups had access to their dams’ milk (DM). The kids from the first treatment group had free access to CF containing alfalfa (CFA) while those from the second group had free access to CF without alfalfa. The third treatment group (control) had access to their DM only. All three groups were kept isolated from the dams from 800 to 1200 h and from 1400 to 1800 h while having access to CF.

**Results:**

Total weight gain and average daily gain of kids from CFA group (11.2±0.36 kg, 145.2±4.64 g) was significantly higher (p<0.05) than kids from CF (7.9±0.49 kg, 102.9±6.43 g) and DM (5.5±0.43 kg, 71.1±5.56 g) groups. The weaning weight of kids from CFA group (15.6±0.39 kg) was significantly higher (p<0.05) than those from CF (12.1±0.56 kg) and DM (9.9±0.59 kg) groups.

**Conclusion:**

This result shows that supplementation of CF combined with alfalfa from birth to weaning enhances growth performance of cross-bred Boer goat kids.

## Introduction

Growth traits are important factors influencing profitability for any goat meat producing enterprise. Rapid growth during early life can minimize the cost of rearing and thus provide more profit to the farmer [1]. The pre-weaning performance of kids provides a stage upon which post-weaning performance is built [2]. The birth weight and early growth rate of animals are determined not only by the genetic potential but also by maternal and environmental factors [3]. Since milk production in goats peaks within 2-3 weeks after parturition and then declines rapidly to a low level by 8-10 weeks after parturition [4], higher growth performance of kids cannot only be sustained by milk supply from their dams. Goat kids from single litters have been shown to have better growth performance than those from multiple litters. Similarly, concentrate supplementation in kids has been shown to improve greatly growth performance especially when milk yield is low [5]. Thus, supplementation of creep feed (CF) is, therefore, necessary to maintain and enhance the pre-weaning growth performance of goat kids. Creep feeding can increase pre-weaning weight gain especially for kids reared as twin or triplet and kids will reach early target market weights, thus increasing net profit for the farmer [6].

Globally, the need to enhance ruminant production by improving carcass quality and milk production has intensified efforts of ruminant researchers towards formulating improved ruminant feeds. Increased average daily gain (ADG) and carcass weight have been reported in goat kids and 10 month old young Boer goats fed alfalfa when compared with grass fed counterparts [7]. However, in a similar study, partial to complete replacement of alfalfa in Shami goat kids did not adversely affect the growth performance and carcass characteristics [8]. In yet another study, increased body and carcass weights in kids fed alfalfa and red clover was observed after four months in comparison to those fed orchard grass, however, dressing percentage was found to be lower in alfalfa fed kids [9]. In Malaysia, the goat population is 505,034 heads [10] but the practice of creep feeding to goat kids is very rare because of unavailability of local CF in commercial quantities. Furthermore, farmers are unaware of manipulating creep feeding regiments to goat kids during weaning in order to improve growth performance. Although considerable works on the effect of creep feeding have been done in calves and lambs, there is still a paucity of literature regarding the effects of CF supplementation with alfalfa on the pre-weaning growth performance of goat kids. Therefore, this study was conducted in order to evaluate the effect of supplementation of CF with or without alfalfa on the pre-weaning growth performance of goat kids.

## Materials and Methods

### Ethical approval

The study protocol followed the ethical guidelines on the proper care and use of animals and had been approved by the Institutional Animal Ethics Committee Universiti Malaysia Kelantan.

### Experimental site

This experiment was carried out at a commercial goat farm from May to September, 2012. The farm (KLAS Farm) is situated at North Latitude 2.39545 and East Longitude 102.11482, Melaka State, Peninsular Malaysia.

### Experimental animals and design

A total of 48, 7 days old, single born, male and female Boer crossbred kids were randomly assigned into three treatment groups by using research randomizer. Each group comprised of eight male and eight female kids having an average weight of 4.4±0.09 kg. The kids in all three groups were allowed to suckle their mothers’ milk until they were weaned at day 84. Kids stayed together with their mothers from 1800 h to 0800 h and from 1200 h to 1400 h throughout the experimental period. Kids in group A were fed dam’s milk (DM)+CF containing alfalfa hay (*Medicago sativa*), (CFA). Group B were fed DM+CF without alfalfa. Group C were only given access to the DM and was considered as the control group.

### CF formulation

CF (mash form) was formulated based on the nutrient requirements of goats (NRC, 1981) using locally available raw materials and alfalfa hay. Samples of CF were sent to the reference laboratory (UNIPEQ, Kuala Lumpur) for the chemical analysis of crude protein, crude fat, carbohydrate, ash, moisture, metabolizable energy, crude fiber. The feed compositions and chemical analysis of formulated CF are shown in Table-1.

**Table-1:** Ration composition and chemical analysis of creep feeds fed to CFA and CF groups.

Creep feed composition %	Groups	

	CFA	CF
Ration composition		
Soya hull	32.0	34.0
Corn	33.0	37.0
Palm Kernal cake	17.0	22.0
Alfalfa hay	11.0	-
Molasses	5.0	5.0
Ammonium chloride	0.5	0.5
Sodium chloride	0.5	0.5
Calcium carbonate	1.0	1.0
Chemical composition		
Crude protein (g/100 g)	14.0	14.2
Crude fat (g/100 g)	5.0	4.4
Carbohydrate (g/100 g)	64.2	62.7
Ash (g/100 g)	4.6	5.5
Moisture (g/100 g)	12.2	13.2
*Metabolizable energy (kcal/kg)	3162	3066
Crude fiber (g/100g)	5.2	0.3

%Total carbohydrate=100–(% Ash+% Moisture+ % Protein+% Fat),

* Calculation by factor, CFA=Creep feed containing alfalfa, CF=Creep feed without alfalfa

### Feeding regiment

The dams and their single born kids were kept together in individual pens (4 feet×5 feet), where the kids had free access to the DM, except at the time of feeding. During feeding, the kids from all three groups were separated from the dams and kept in the feeding compartments (12 feet×12 feet) adjacent to their pens. The treatment groups (CFA and CF) were fed according to their assigned feeds, twice a day; (800-1200 h and 1400-1800 h; Table-2) while the control group DM was only given water. Dams were fed twice a day; from 0800 to 1200 h and from 1400 h to 1800 h. All the left over feed from the dams’ feeding troughs were removed at the end of their feeding time before the kids were mixed with their dams. The kids from CFA and CF were fed CF (5% of body weight) on the dry matter basis. Feed were renewed daily. Clean water was made available to all the groups throughout the study period. Average overall feed consumption was calculated based on the left over feed in the feeding troughs

**Table-2:** The feeding program of the experimental groups; CFA, CF and DM.

Time (h)	CFA	CF	DM
800-1200	Creep feeding	Creep feeding	Water only
1200-1400	With mother	With mother	With mother
1400-1800	Creep feeding	Creep feeding	Water only
1800-800	With mother	With mother	With mother

CFA=Creep feed containing alfalfa, CF=Creep feed without alfalfa, DM=Dam’s milk

### Determination of body weights and growth rates

The kid’s body weights were taken and recorded every 7 days before feeding (8 am) for 12 weeks by using a calibrated hanging weigh balance (SALTER, Model 235; Max-25 kg, d-100 g).

The growth rate of kids in all treatment groups was calculated using the following formula.


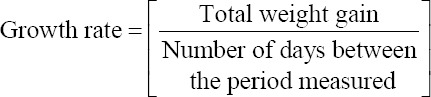


### Statistical analysis

Data collected from growth performance were presented as mean±standard deviation and analyzed using one-way ANOVA, SPSS (version 19). Differences between means were tested for statistical significance with Tukey test with a significant level of p<0.05.

## Results

The results show that the feed consumption of kids from CFA was about 290 g/day, while that of CF was about 230 g/day.

### Growth performance (7-28 days)

**Figure-1:**
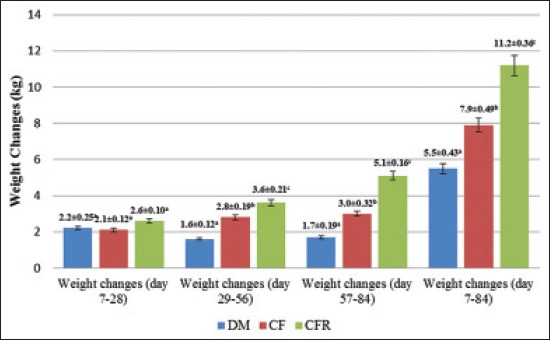
Weight changes of experimental kids from dams’ milk, creep feed and creep feed containing alfalfa treatment groups at different age periods Values in the figures were means±standard error of each treatment group (n=16). Different superscripts above the bars in each age period significantly differ (p<0.05).

Kids in the CFA treatment group were the first to take the CF. By day 28, kids from the CFA group began nibbling at the CF, but they were very selective as they consumed the cut alfalfa hay first. This feeding behavior was not observed in the CF group. The average daily weight gains of CFA, CF and DM groups were 122, 97.6 and 102.7 g, respectively (Table-3). The weight gains were not significantly different between the groups (p>0.05). Figure-1 shows the growth performance of kids from during the three selected time periods; day 7 and 28, day 29-56 and day 57-84 in the three treatment groups.

**Table-3:** ADG (g/day) of kids from DM, CF and CFA treatment groups (n=16) at different age periods.

ADG	Mean±SE		

	CFA	CF	DM
ADG (day 7-28)	122.0±4.89^a^	97.6±5.83^a^	102.7±11.73^a^
ADG (day 29-56)	129.0±7.66^a^	101.3±6.63^b^	58.0±4.27^c^
ADG (day 57-84)	178.0±5.76^a^	108.3±11.34^b^	60.5±7.00^c^
ADG (day 7-84)	145.2±4.64^a^	102.9±6.43^b^	71.1±5.56^c^

a,b,c Means with different superscripts with a row are significantly different (p<0.05), CFA=Creep feed containing alfalfa, CF=Creep feed without alfalfa, DM=Dam’s milk, SE=Standard error, ADG=Average daily gain

### Growth performance (29-56 days)

At 35 day, all kids in the CFA group ate the CF but selective feeding was still observed among the group. Only a few kids in the CF group started to show interest in the CF at this stage. However, by day 42, all kids in the CF group ate the CF. Kids from the CFA group had the highest weight gains during this period (3.6 kg), whereas kids from the DM group had the least weight gain (1.6 kg). The weight gains were significantly different among the three groups (p<0.05) (Table-3). The growth performance of kids between day 29 and 56, in the three treatment groups are summarized in Figure-1.

### Growth performance (57-84 days)

On day 57, selective feeding in the CFA group was no longer prominent. Better growth performance was also observed during this period compared to earlier weeks. The weight gain for the CFA kids was significantly higher than those in the CF and DM groups (p<0.05). The average daily gain (ADG) for the CFA group was the highest (179 g/day) while for CF and DM group were 108.3 g/day and 60.5 g/day respectively (Table-3). The ADG of the three treatment groups were significantly different each other (p<0.05).

### Overall growth performance (7-84 days)

There was no kid motility in all the treatment groups during the experimental period. Average dry matter intake (DMI) for the 12 weeks period was greater for CFA (290 g/day) than CF (230 g/day). The weaning weights of kids in the CFA group were significantly heavier than those of kids in the CF and DM groups (p<0.05; Table-4): on average 3.3 kg heavier than the CF and 5.7 kg heavier than the DM group.

**Table-4:** Average weights of kids from CFA, CF and DM groups at different periods of the experiment (n=16).

Weight (kg)	Mean±SE		

	CFA	CF	DM
Birth weight	3.15^a^±0.92	3.06^a^±0.11	3.40^a^±0.11
Weight at day 7	4.4^a^±0.10	4.2^a^±0.15	4.5^a^±0.18
Weight at day 28	7.0^a^±0.14	6.6^a^±0.82	6.5^a^±0.34
Weight at day 56	10.6^a^±0.30	9.1^b^±0.38	8.2^b^±0.39
Weaning weight at day 84	15.6^a^±0.39	12.1^b^±0.56	9.9^c^±0.59

a,b,c Means with different superscripts with a row are significantly different (p<0.05), CFA=Creep feed containing alfalfa, CF=Creep feed without alfalfa, DM=Dam’s milk, SE=Standard error

### Growth performance in male and female kids

The overall weight changes and ADG of male and female kids in all the groups are summarized in Table-5. The average body weights and daily weight gains increased significantly (p<0.05) in the CFA group in both male and female kids during the course of the study (7-84 days). However, these two parameters were not significantly (p>0.05) different in the CF and DM groups of male kids during the same period, while in female kids, there was a significant difference (p<0.05) between the CF and DM groups.

**Table-5:** Weight changes and ADG of male and female kids from CFA, CF and DM groups (n=8).

Item	Means±SE		

	CFA	CF	DM
Weight increase of male kid (kg)			
Day 7-28	2.5^a^±0.16	2.0^a^±0.21	2.7^a^±0.39
Day 28-56	3.8^a^±0.37	2.8^ab^±0.33	1.8^b^±0.20
Day 56-84	5.1^a^±0.24	2.7^b^±0.50	1.7^b^±0.31
Day 7-84	11.4^a^±0.63	7.5^b^±0.82	6.2^b^±0.77
Weight increase of female kid (kg)			
Day 7-28	2.7^a^±0.13	2.1^ac^±0.15	1.6^c^±0.16
Day 28-56	3.4^a^±0.22	2.9^a^±0.19	1.5^b^±0.12
Day 56-84	4.9^a^±0.23	3.4^b^±0.38	1.7^c^±0.27
Day 7-84	11.0^a^±0.38	8.3^b^±0.57	4.8^c^±0.22
Daily gain of male kids (g/day)			
Day 7-28	117.3^a^±7.79	95.9^a^±9.79	127.4^a^±18.96
Day 28-56	137.1^a^±13.05	100.9^ab^±11.84	62.9^b^±7.27
Day 56-84	181.3^a^±8.61	96.0^b^±17.79	62.1^b^±10.92
Day 7-84	147.7^a^±8.14	97.7^b^±10.66	80.2^b^±10.12
Daily gain of female kids (g/day)			
Day 7-28	126.8^a^±5.96	99.4^b^±6.99	77.9^b^±7.88
Day 28-56	120.9^a^±7.91	101.8^a^±6.94	53.1^b^±4.29
Day 56-84	176.3^a^±8.16	120.5^b^±13.63	58.9^c^±9.50
Day 7-84	142.7^a^±4.93	107.9^b^±7.50	61.9^c^±2.89

*a,b,c Values with different superscripts in the same row differ significantly (p<0.05), CFA=Creep feed containing alfalfa, CF=Creep feed, DM=Dam’s milk, ADG=Average daily gain

## Discussion

One of the most influencing factors of milk yield in the doe is the type of birth; single, twin or triplet [11]. In this study, kids from all the three groups were single born and thus there might not be much variation in milk intake between the experiment groups. It was observed that the ADG of kids from CFA group was significantly (p<0.05) greater than ADG of kids from CF and DM groups during the 12-week period. This agrees with the findings of Goetsch *et al*. [5], which states that kids from single litters perform better than those from multiple litters and concentrate supplementation increases postweaning body weight and growth performance, especially if milk yield is low. We recorded an average ADG of 145.2±4.64 g in the CFA group, which was higher than ADG reported by Browning and Leite-Browning [12] in which crossbred Boer kids had ADG of 116.9 g after 3 months. This shows that creep feeding practiced in intensive management system enhances better growth performance in the kids than in semi-intensively managed systems.

We noticed that in week 4, kids from CFA group started nibbling small amounts of feed where alfalfa was selected first. This agrees with findings of Luo *et al*. [13], who reported that kids did not consume significant amounts of starter diet until when they have reached 5 weeks of age. In this experiment, kids from CF group had a lower intake of CF than CFA kids. This may be due to the absence of alfalfa in the feed combination. In a related study in calves, Coverdale *et al*. [14] found that feeding 7.5-15% of ground grass hay improved starter feed intake and daily weight gain of calves as compared to feeding coarse or ground grain diets only.

The difference in forage to concentrate ratio (11:89 in CFA and 0:100 in CF) may also have attributed to the higher growth performance of CFA group. Haddad [15] found that low forage to concentrate ratio had increased DMI than higher forage to concentrate ratio. On the other hand, shorter particle length of the roughage can increase DMI [16]. This may also be one of the factors why kids in the CFA group in this study consumed more feed and gained more weight than those from CF and DM groups. The kids from DM group gained least as they relied on their DM only. Bhatt *et al*. [17] reported that pre-weaning parameters such as body weight, ADG and feed conversion ratio were higher in lambs fed CF and milk replacer supplementation. This concurs with our findings in this study and also with other studies [18,19], which showed that supplementation of different pastures during weaning increase ADG of kids and lambs. Since kidding season has an influence on the total weaning weight of kids as reported by Andries [20], availability of pasture may hinder creep roughage availability in some areas. A related study by Borges *et al*. [21] found out that CF supplementation from birth produced better weight gains than when started at 20 or 40 days after birth. Even though, the farmer has the different option of forage to choose from, alfalfa has been shown previously to produce good results with relation to digestive organ weight and development in Rasa Aragonesa lambs in Spain [22]. Furthermore, the length of lactation was shown to enhance rumen development especially in lambs fed concentrates, but was not significantly affected by alfalfa grazing regardless of access to milk [23]. However, no similar study was undertaken in goat kids in Malaysia. In a related study, Mimosa leaves were shown to be preferred by goat kids when combined with several other forages [19]. The practice of CF may not be economically viable especially in small holder farmer lots that also use the DM for consumption. Hence is more practicable in commercial systems [24].

## Conclusion

This study showed that feeding suckling goat kids a combination of CF and alfalfa produced better weight gain and growth performance than feeding CF alone or DM alone.

## Authors’ Contributions

NNH conducted the study and was supervised by ATK, NNH, TK and MAKGK. YA, JFFA and MAML revised the manuscript draft and statistical analysis. All authors have read and approved the manuscript.
